# Cytotoxicity of PEG-Coated Gold and Gold–Iron Alloy Nanoparticles: ROS or Ferroptosis?

**DOI:** 10.3390/nano13233044

**Published:** 2023-11-29

**Authors:** Clara M. G. de Faria, Michael Bissoli, Riccardo Vago, Antonello E. Spinelli, Vincenzo Amendola

**Affiliations:** 1 Department of Chemical Sciences, University of Padova, Via Marzolo 1, I-35131 Padova, Italy; claramaria.goncalvesdefaria@unipd.it (C.M.G.d.F.); michael.bissoli@unipd.it (M.B.); 2 Urological Research Institute, Division of Experimental Oncology, IRCCS San Raffaele Scientific Institute, I-20132 Milan, Italy; vago.riccardo@hsr.it; 3 Experimental Imaging Center, IRCCS San Raffaele Scientific Institute, I-20132 Milan, Italy; spinelli.antonello@hsr.it

**Keywords:** nanomedicine, laser ablation in liquid, nanoalloys, cytotoxicity, biocompatibility

## Abstract

Nanomedicine relies on the exploitation of nanoscale constructs for therapeutic and diagnostic functions. Gold and gold–iron alloy nanoparticles (NPs) are two examples of nanomaterials with favorable features for use in nanomedicine. While gold NPs have been studied extensively in the last decades, they are not biodegradable. Nonetheless, biodegradation was recently observed in gold alloys with iron obtained using laser ablation in liquid (LAL). Hence, there is a significant interest in the study of the biological effects of gold and gold–iron alloy nanoparticles, starting from their tolerability and cytotoxicity. In this study, these two classes of NPs, obtained via LAL and coated with biocompatible polymers such as polyethylene glycol, were investigated in terms of their cytotoxicity in fibroblasts, prostate cancer cells (PC3) and embryonic kidney cells (HEK). We also explored the effects of different synthetic procedures, stabilizing additives, and the possible mechanisms behind cell mortality such as the formation of reactive oxygen species (ROS) or ferroptosis. NPs larger than 200 nm were associated with lower cell tolerability. The most tolerable formulations were pure PEG-Au NPs, followed by PEG-Au–Fe NPs with a hydrodynamic size < 50 nm, which displayed a toxicity of only 20% in fibroblasts after 72 h of incubation. In addition, tumor cells and highly proliferating HEK cells are more sensitive to the NPs than fibroblasts. However, a protective effect of catalase was found for cells incubated with PEG-Au–Fe NPs, indicating an important role of hydrogen peroxide in alloy NP interactions with cells. These results are crucial for directing future synthetic efforts for the realization of biocompatible Au NPs and biodegradable and cytocompatible Au–Fe alloy NPs. Moreover, the correlation of the cytocompatibility of NPs with ROS and ferroptosis in cells is of general interest and applicability to other types of nanomaterials.

## 1. Introduction

Since its proposal in the late 1960s, nanomedicine has become a rapidly developing field due to its great potential to overcome long-known challenges in the diagnosis, monitoring, control, prevention and treatment of diseases [1]. Nanomaterials were developed as drug delivery vehicles, contrast agents and diagnostic devices, showing exceptional features in their interactions with biomedical systems such as the enhancement of standard imaging and therapeutic functions, and even the target-oriented precise delivery of drugs [2,3].

Several nanomaterials have been proposed to date, with a variety of different features and compositions. For instance, lipidic nanoparticles have enhanced the delivery of drugs to cancer tissues [4], while inorganic compounds such as silicon nanoparticles [5] have original properties not found in typical organic drugs. Semiconductor quantum dots are bright labels for bioimaging [6], and oxide compounds such as ceria have good performances for X-ray computed tomography (CT) [7].

In particular, Au and Au-based NPs have played a prominent role in this context due to the biocompatibility and stability of this metal and its ease of functionalization with thiolated compounds [8,9]. Au NPs have been widely investigated as drug delivery carriers, thanks to the possibility of acting on their coating for adding new chemical functionalities, finding applications in cardiovascular diseases by antioxidative/anti-inflammatory properties [10], multiple sclerosis, diabetes, Parkinson’s, Alzheimer’s and other infectious diseases [8]. Au NPs are also considered as sensitizers for photothermal therapy and X-ray radiotherapy and as contrast agents for CT [11,12,13].

Iron-based nanoparticles also display a combination of interesting magnetic properties with a high biocompatibility that allows the improvement of diagnostic systems such as magnetic resonance imaging (MRI), drug delivery, magnetic separation, cell proliferation and tissue repair, as well as the localized and controlled use of hyperthermia in therapeutics [14,15,16]. They can be iron oxides and ferrites, as well as metal alloy nanoparticles like FePt and FeCo [17,18]. Recently, Au–Fe nanoalloys synthesized using a laser ablation in liquid (LAL) route displayed spontaneous size reduction and biodegradation in the physiological environment after use, allowing clearance from the body on the short/medium term while being compatible also with surface coatings with thiolated compounds [19]. This is a relevant feature compared to pure Au NPs because large particles are associated with increased tumor retention due to poor lymphatic drainage, but reside in the liver and spleen for a very long time, with only 9% of the pure gold NPs being eliminated in 6 months. Instead, smaller NPs further increase tumor penetration and allow clearance from major organs over the medium term [20,21,22]. In this context, the Au–Fe nanoalloys behaved as nanoparticles with transformable size, initially 30–200 nm, that is further reduced to <10 nm to combine the advantages of the two regimes [19]. Furthermore, due to their composition, the Au–Fe alloys have the ability to act as multimodal contrast agents for MRI and CT, and can be studied as possible sensitizers for radiotherapy, one of the major therapeutic modalities for cancer treatment with about half of all oncological patients receiving it at some point of the treatment [13,23]. The Au–Fe nanoalloys can further be decorated with ligands to better penetrate cell membranes or stromal barriers, thus enabling various therapeutic and diagnostic possibilities [8,9,24]. LAL played a crucial role in the study and realization of these Au–Fe nanoalloys, because they are metastable phases of difficult synthesis and surface functionalization with other methods. Indeed, the easy surface functionalization and the low cost are also advantages of the LAL synthesis of Au NPs [19,25,26].

Despite its wide potential, the translation of nanomedicine into routine clinical procedures has yet to be achieved [20,23,27]. Some of the main challenges that must be overcome are the poor clearance, low specificity with consequently poor accumulation in the target site, and unclear effects of the nanomaterials on the immune system [20,23,28]. Regulatory agencies are increasingly focused on the safety of nanomedicines, with expert groups providing scientific information about nanomedicines and developing guidelines. One of the potential issues being evaluated is the biopersistence of these nanomedicines, i.e., their presence in specific organs for long periods of time [27]. Biopersistence can be due to a variety of factors, including the size, shape and surface properties of the nanomaterial, thus being important in the development of nanomedicines. The potential long-term effects of the biopersistence of nanomedicine on human health are toxicity, inflammation and alteration of neurotransmitters, but other effects not yet fully understood are also possible [29].

To overcome these challenges, exploring the impact of NPs in vitro is the starting point for their optimization and exploitation for theragnostics. Hence, in this study, we screened the in vitro toxicity of a panel of polyethylene glycol (PEG)-coated Au–Fe NPs candidates produced via LAL and of the reference PEG-coated Au NPs. Different procedures for the coating and purification of the NPs were tested. PEG was used also in combination with a silica coating, which is often applied for the stabilization of iron-based NPs, to seek an increase in the stability in aqueous solution and in the surface area of the NPs, which is beneficial for antibody conjugation. Another combination consisted of PEG with poly(lactic-co-glycolic acid) (PLGA), an FDA-approved polymer widely used in nanomedicine due to its biocompatibility, prolonged resistance time and controlled release properties [30]. Furthermore, silica and PLGA coatings can be used to load drugs or imaging agents. The goal was to select the most promising formulation for future application in cancer therapy via X-ray radiosensitization and chemiodynamic effects guided by imaging techniques.

## 2. Materials and Methods

### 2.1. NPs Synthesis

All of the NPs were produced via LAL by focusing with a f 100 mm lens the 1064 nm (6 ns, 50 Hz) laser pulses at a fluence of 14 J/cm^2^ on a bulk target (either Au 99.99% pure or Au–Fe 25–75 99.9% pure from Mateck GmbH, Juelich, Germany) dipped in a liquid solution. To homogeneously ablate the target, the ablation cell was mounted on a motorized XY scanning stage (Standa, Vilnius, Lithuania) controlled with a 2-axis stepper and a DC motor controller. Each synthesis lasted for 3 h and was repeated depending on the quantity of NPs required.

The polyethylene glycol (PEG)-coated gold NPs (PEG-Au NPs) were synthesized via LAL in 2 × 10^−4^ M NaCl solution in distilled water. Then, a mixture of mPEG-SH (5 kDa, Laysan bio, Arab, AL, USA) and SH-PEG-COOH (3 kDa, Rapp Polymere, Tuebingen, Germany) with a 1:1 molar ratio and final concentration of 0.087 mg/mL was added to the colloid. After 20 min in a bath sonicator and 3 h at room temperature, the colloid was dialyzed with concentration membranes (Vivaspin 20, cutoff 10 kDa, Sartorius, Goettingen, Germany) and resuspended in distilled water.

**Scheme 1:**
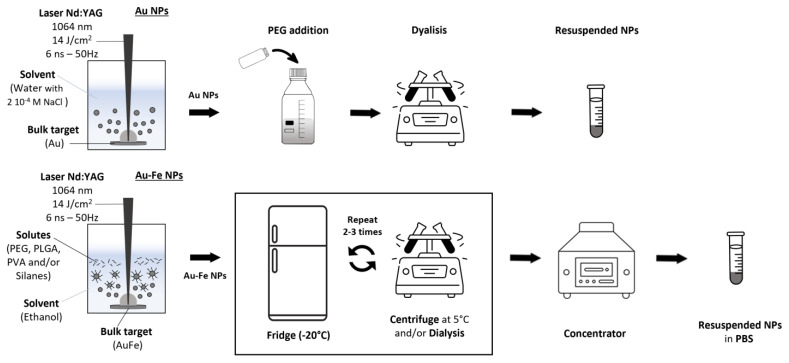
General procedure for the synthesis of PEG-Au and PEG-Au–Fe NPs.

The PEG-coated Au–Fe NPs were synthesized and subsequently purified following a previously published procedure [19]. Different types of PEG-coated Au–Fe NP samples were produced and tested as described below and summarized in Scheme 1.

The PEG-coated Au–Fe n1 NPs (PEG-Au–Fe n1) were synthesized via LAL in ethanol (HPLC grade, Sigma Aldrich Burlington, MA, USA) containing a mixture of mPEG-SH and SH-PEG-COOH with a 1:1 molar ratio and final concentration of 0.087 mg/mL, in an Ar atmosphere and under magnetic stirring. Then, the colloid was kept at −20 °C overnight and centrifuged at 1000 rcf for 1 h at 5 °C. The procedure was repeated three more times, washing the sediment with acetone (ultrapure, Sigma-Aldrich) at 1000 rcf for 1 h at 5 °C. Finally, the sediment was dried at room temperature with an Eppendorf Concentrator Plus to remove all of the organic solvent, and stored in a freezer until use by addition of phosphate buffer saline (PBS) solution and redissolution with a bath sonicator at 20 °C for 20 min.

The PEG-coated Au–Fe n2 NPs (PEG-Au–Fe n2) were synthesized with the same procedure as the n1 sample. Then, the colloid was kept at −20 °C overnight and centrifuged once at 1000 rcf for 1 h at 5 °C. The sediment was dried at room temperature with an Eppendorf Concentrator Plus to remove all of the organic solvent. Subsequently, the NPs were redispersed in 2 mL of PBS and dialyzed with concentration membranes (Vivaspin 2, cutoff 50 kDa), washing three more times with distilled water. Finally, the sediment was dried at 30 °C with an Eppendorf Concentrator Plus to remove all of the organic solvent and stored in a freezer until use by addition of PBS solution and redissolution with a bath sonicator at 0 °C for 20 min.

The PEG-coated Au–Fe n3 NPs (PEG-Au–Fe n3) were synthesized starting from the PEG-Au–Fe n2 sample, which was redispersed in a 1:4 vol:vol mixture of ethanol and methanol (both HPLC grade, from Sigma Aldrich) and washed via centrifugation two times at 1000 rcf for 1 h at 5 °C. Finally, the sediment was dried at room temperature with an Eppendorf Concentrator Plus to remove all of the organic solvent and stored in a freezer until use by addition of PBS solution and redissolution with a bath sonicator at 20 °C for 20 min.

The PEG-coated Au–Fe n4 NPs (PEG-Au–Fe n4) were synthesized with the same procedure as the n1 sample. Then, the colloid was kept at −20 °C overnight and centrifuged once at 1000 rcf for 1 h at 5 °C. The procedure was repeated two more times with a 1:4 vol:vol mixture of ethanol and methanol (both HPLC grade, from Sigma Aldrich). The sediment was dried at room temperature with an Eppendorf Concentrator Plus to remove all of the organic solvent. Subsequently, the NPs were redispersed in 2 mL of PBS with 0.1 mg/mL mPEG-SH 5 kDa and dialyzed with concentration membranes (Vivaspin 2, cutoff 50 kDa), washing three more times with distilled water. Finally, the NPs were dispersed in distilled water and stored at 4 °C until use.

The PEG-coated Au–Fe n5 NPs (PEG-Au–Fe n5) were synthesized with the same procedure as the n1 sample. Then, the colloid was kept at −20 °C overnight and centrifuged once at 1000 rcf for 1 h at 5 °C. The procedure was repeated two more times with acetone (ultrapure, Sigma-Aldrich). The sediment was dried at room temperature with an Eppendorf Concentrator Plus to remove all of the organic solvent. Subsequently, the NPs were redispersed in 2 mL of PBS with 0.1 mg/mL mPEG-SH 5000 Da and dialyzed with concentration membranes (Vivaspin 2, cutoff 50 kDa), washing three more times with distilled water. Finally, the NPs were dispersed in distilled water and stored at 4 °C until use.

The tetraethoxysilane (TEOS)-coated PEG-Au–Fe NPs (TEOS-PEG-Au–Fe a and b) were synthesized via LAL in ethanol (HPLC grade, Sigma Aldrich) containing a mixture of mPEG-SH and SH-PEG-COOH at a 1:1 molar ratio and final concentration of 0.087 mg/mL and 0.0125 mg/mL (sample a) or 0.0627 mg/mL (sample b) TEOS (Fluka, Buchs, Switzerland), in an Ar atmosphere and under magnetic stirring. Then, the colloid was kept at −20 °C overnight and centrifuged once at 1000 rcf for 1 h at 5 °C. The procedure was repeated two more times with a 1:4 vol:vol mixture of ethanol and methanol (both HPLC grade, from Sigma Aldrich). The sediment was dried at room temperature with an Eppendorf Concentrator Plus to remove all of the organic solvent. Subsequently, the NPs were redispersed in 2 mL of PBS with 0.1 mg/mL mPEG-SH 5 kDa and dialyzed with concentration membranes (Vivaspin 2, cutoff 50 kDa), washing three more times with distilled water. Finally, the NPs were dispersed in distilled water, filtered with a 450 nm cutoff cellulose acetate filter (VWR) and stored at 4 °C until use.

The samples embedded in the poly[DL-lactide-co-glycolide] copolymer (PLGA, 50:50 lactide-glycolide ratio, 7–17 kDa, Sigma-Aldrich) were obtained starting from the PEG-Au NPs (PLGA-PEG Au) and the PEG-Au–Fe n1 NPs (PLGA-PEG-Au–Fe n1) samples. The PEG-Au NPs were dried at 30 °C with an Eppendorf Concentrator Plus. The NPs (either PEG-Au or PEG-Au–Fe n1) were dissolved at 0.5 mg/mL in 1 mL of acetone:ethanol 85:15 vol:vol containing 10 mg/mL PLGA. This solution was added dropwise at room temperature to 10 mL of milliQ water (filtered with a 200 nm filter) containing 1 wt% polyvinyl alcohol (PVA, 40 kDa, Sigma-Aldrich), while sonicating with a bath sonicator. The solution was left under sonication for 5 min, and then stirring was maintained at 20 °C overnight under a nitrogen atmosphere to evaporate the organic phase. Subsequently, the colloid was dialyzed three times with distilled water using a concentration membrane (Vivaspin 2, cutoff 100 kDa) to remove the excess PLGA and PVA. Finally, the NPs were filtered with a 450 nm cutoff cellulose acetate filter (VWR) and stored at 4 °C until use.

### 2.2. NPs Characterization

The UV–visible spectra were recorded with a V770 spectrophotometer (JASCO, Easton, MD, USA) using quartz cells with a 2 mm optical path, and by an Avantes (Apeldoorn, The Netherlands) portable spectrometer (AvaSpec-ULS2048CL-EVO) coupled with a deuterium-halogen lamp (AVA-Light-DHc). FTIR was performed with a 1720X FTIR spectrophotometer (Perkin Elmer, Waltham, MA, USA), depositing the dried samples on a KBr window. Dynamic light scattering (DLS) was performed in DTS1070 cells and z-spectroscopy in DTS1070 cuvettes with a Zetasizer Nano ZS (Malvern, Malvern, UK).

TEM analysis was carried out with an FEI Tecnai G2 12 transmission electron microscope operating at 100 kV equipped with a TVIPS CCD camera. Samples were prepared by evaporating the colloids on a copper grid coated with an amorphous carbon holey film.

X-ray diffraction (XRD) analysis was performed with a XPert 3 Powder diffractometer equipped with a Cu tube (40 kV, 40 mA), a BBHD mirror, a spinner and a PlXcel detector (Panalytical, Malvern, UK). The samples were deposed on Si zero-background substrates by drop-casting and drying at room temperature. Crystalline phase identification was executed with a search/match procedure using DIFFRAC.EVA 7 software (Bruker, Billerica, MA, USA) and a COD database, while the diffractograms were analyzed with TOPAS Academic V6 (Bruker AXS). Rietveld refinements were carried out by fitting the background with a Chebychev function, a broad-Gaussian peak due to the amorphous phase and the required phases. Fit indicators Rwp, Rexp and GoF (Goodness of Fit) were used to assess the quality of the refined structural models.

### 2.3. Toxicity Dose–Response Curve

The used cell types were from human bone metastasis of grade IV prostatic adenocarcinoma cells PC3 (CRL-1435, ATCC, Rockwell, MD, USA), human fibroblasts BJ (CRL-2522, ATCC, Rockwell, MD, USA) and human embryonic kidney cells HEK (CRL-1573, ATCC, Rockwell, MD, USA).

In cell viability assessment via colorimetric MTT assay, the cells were seeded in a 96-well plate at a density of 5 × 10^3^ cells/well for fibroblasts and HEK cells and 3 × 10^3^ cells/well for PC3, and placed on an incubator at 37 °C and a 5% CO_2_ atmosphere overnight. After medium removal, 100 µL of fresh medium with nanoparticles at various concentrations was added to each well, and the plates were incubated for 48 h or 72 h, depending on the experiment. The concentrations of the NPs were set in agreement with those of previous studies regarding the cytotoxicity of Au and Au–Fe NPs [26,31]. Then, the medium was removed, the wells are washed with phosphate sulfate buffer, and 3-(4,5-dimethyl-thiazol-2-yl)-2,5-diphenyl-tetrazolium bromide (MTT) was added at a concentration of 0.5 mg/mL into the medium. After 2 h of incubation, the MTT was removed, and formazan crystals were dissolved in 100 µL of dimethyl sulfoxide (DMSO); the absorbance was read at 590 nm in a plate reader. The viability was calculated as a ratio of the absorbance of the well divided by the mean absorbance of the control group. Positive control was performed with water instead of cell culture medium and yielded absorbance maximum values corresponding to 5% viability. Each condition was prepared in triplicate, and at least two independent experiments were performed.

The concentration of NPs that yielded a cell viability of 50% (IC50) was calculated after fitting the experimental data to a sigmoid dose–response curve. The formula used to fit the curves with the two parameters (*IC*50 and *Hill coefficient*) is as follows:

Viability (C)=Max−Min1+CIC50Hill coefficient 

where *Max* is set as 1 (100% of viability) and *Min* is 0 (0% viability), and *C* represents the nanoparticle concentration. The “IC50 Calculator” from AAT Bioquest was used for fitting, using the two-parameter mode [32].

### 2.4. Crystal Violet Assay

The crystal violet (CV) assay was carried out along with an MTT assay at the same conditions, in a mirrored plate, with identical steps until the staining, where the CV wells were washed twice with PBS and incubated for 10 min with a CV solution (11% methanol, 0.1% crystal violet). After the CV solution was removed, the wells were washed once with PBS; 100 µL of DMSO was added to solubilize the crystal violet. The absorbance at 570 nm was read with a multiplate reader.

### 2.5. ROS Scavengers

A volume of 50 µL of freshly prepared n-acetylcysteine (NAC, A9165 Sigma-Aldrich, Burlington, MA, USA), catalase (CAT, C1345 Sigma-Aldrich, Burlington, MA, USA) and dimethyl sulfoxide (DMSO, A3672 PanReac AppliChem, Monza, Italy) solutions in medium was added to the wells after medium removal. The pH of the NAC stock solution was corrected with NaOH to 7.4. After 1 h of incubation, 50 µL of medium with NPs was added to each well, resulting in a final concentration of 10 mM for NAC, 500 µg/mL for CAT and 1% (*v*/*v*) for DMSO, and 150 µg/mL for PEG-Au or PEG-Au–Fe n1, and 50 µg/mL for TEOS-PEG-Au–Fe b. At the end of the incubation period of 48 h at 37 °C, the wells were washed and an MTT assay was performed to evaluate the cell viability. Three independent experiments were performed with PC3, HEK and BJ cells.

### 2.6. Ferroptosis Assay

The viability assay with MTT as described in the previous section was performed for the PEG-Au–Fe, PEG-Au–Fe n1 and TEOS-PEG-Au–Fe b (the latter filtered with a 200 nm filter) samples at 50 µg/mL. A co-incubation with ferrostatin-1 (Fer-1) at 2 µM was performed and compared to the groups without Fer-1. All three cell lines were tested, and three independent experiments were performed.

### 2.7. Statistical Analysis

The results are displayed as means ± SE of three or two independent experiments. In the experiments with *n* = 3, a normality test was performed prior to the one-way ANOVA analysis to identify statistical significance among the data with *p* = 0.05. All processing was performed using Origin software (OriginLab 2022b, Northampton, MA, USA).

## 3. Results

### 3.1. Characterization of NPs Samples

The Au and Au–Fe NPs were obtained via LAL because of its convenient features to produce metal and alloy colloids [19,25,33,34,35,36], as well as its self-standing procedure and low cost [37,38,39]. Both types of NPs were coated with PEG (5000 Da), and then subjected to various purification protocols as described in the Methods section. In particular, the LAL of Au–Fe NPs was performed in a liquid solution of PEG to obtain the coating and stabilization of the nanoalloys in situ. The cleaning protocols were designed to remove the excess polymer and other low-density components soluble in polar organic solvents at low temperature (−20 °C or 5 °C), i.e., in conditions where the PEG-coated Au–Fe NPs are not stable and can easily be separated from the liquid phase by centrifugation. However, the procedure was also varied in the type of coating agents and cleaning steps used for the removal of polymer and iron-based compounds, seeking a possible improvement in the biocompatibility of the final products.

**Figure 1:**
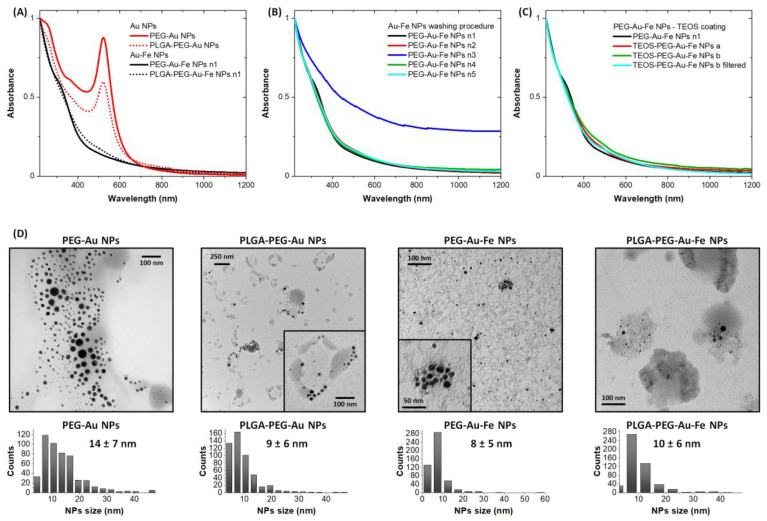
(**A**–**C**) Optical absorption spectra of Au and Au–Fe NPs: (**A**) PEG-Au and PEG-Au–Fe n1 samples before and after encapsulation in PLGA; (**B**) comparison of PEG-Au–Fe samples from n1 to n5, obtained through different washing protocols; (**C**) PEG-Au–Fe samples coated with TEOS. All spectra were normalized at 220 nm for a clearer comparison. (**D**) Representative TEM images of PEG-Au and PEG-Au–Fe n1 samples before and after encapsulation in PLGA. Note the PLGA samples were filtered with a 450 nm cutoff before analysis and use. Size histograms for each sample are also shown.

The optical spectrum of the PEG-Au NPs (Figure 1A) shows the sharp plasmon band at 520 nm that is typical of these nanostructures when they have a spherical shape and are not aggregated. Conversely, the absorption spectrum of the PEG-Au–Fe n1 NPs does not show a clear plasmon band, but only a flat absorption in the visible region and an edge below 400 nm contributed by the interband transitions of the alloy NPs and optical excitations in the noncrystalline iron oxide compounds remaining in the sample [19]. This is quite apparent by the comparison of the TEM images of the PEG-Au and PEG-Au–Fe n1 NPs (Figure 1D), indicating that the average size of the NPs is on the order of 10 nm. The color of the PEG-Au solution was purple red, and that of the PEG-Au–Fe NPs solution was brown, as a consequence of the diversity in the optical absorption spectra.

The Au and Au–Fe NPs were also encapsulated in PLGA to modify the agglomeration of particles in the nanoconstructs. This is especially interesting in the case of the pure gold formulation, which initially consists of individual nanospheres and, after encapsulation, appears as groups of nanospheres in the polymeric matrix (see TEM images in Figure 1D). The PEG-Au–Fe n1 NPs already appeared to be clustered in the TEM image; therefore, their inclusion in the PLGA matrix does not significantly change this feature, which is crucial for the interaction with cells in the in vitro tests.

The presence of iron byproducts in the Au–Fe sample may be dependent on the purification process; therefore, various approaches were tested (PEG-Au–Fe NP samples n2–5) and the effects were evaluated in the subsequent in vitro tests. The optical properties of the PEG-Au–Fe NP samples n1, n2, n4 and n5 remained almost identical (Figure 1B), indicating that few changes were made to the structural composition of the final products. This is not the case of the PEG-Au–Fe n3 NP sample, which was evidently aggregated and unstable in the liquid solution.

Also, the coating of the Au–Fe NPs with both PEG and silanes (TEOS) was attempted (Figure 1C). In fact, iron at the surface of the Au–Fe nanoalloys undergoes oxidation [19], and iron oxide is relatively inert to the reaction with biocompatible polymers, usually not allowing the formation of strong covalent bonds with these stabilizing compounds. However, the binding of iron oxide surfaces with polymers can be improved by coating with a thin layer of silica [40]. TEOS is a precursor for silica that is widely used thanks to its silane groups; strong M-O-Si covalent bonds (M = metal ion) are possible [41], forming a network where polymeric chains can also be embedded [40,41]. Furthermore, by setting the amount of TEOS used, the thickness of the coating can be adjusted [40,41,42]. In the experiment, the optical spectra of the a and b TEOS-PEG-Au–Fe NPs indicate an increase in the absorption in the visible and near infrared range, which can be due to the encapsulation of Au–Fe NPs into a silane network, leading to larger constructs compared to the n1 sample.

### 3.2. Assessment of NPs Toxicity

**Figure 2:**
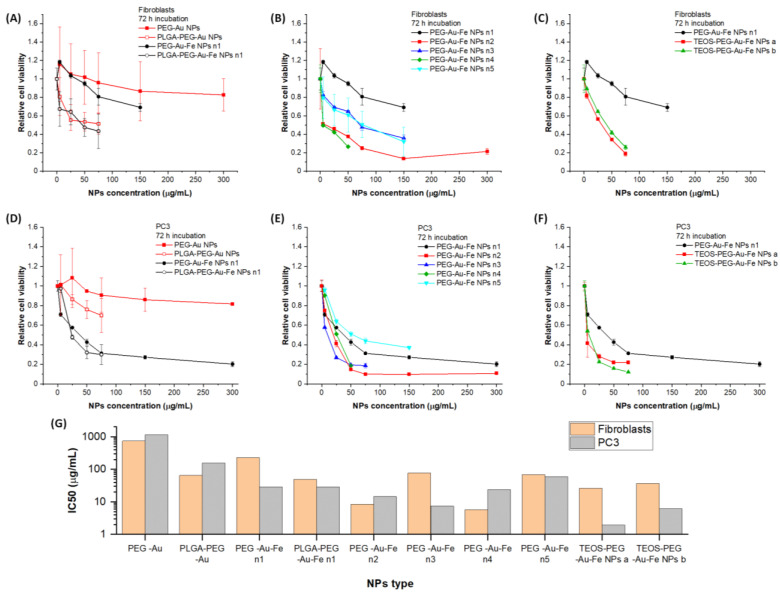
Viability results of fibroblasts (**A**–**C**) and PC3 (**D**–**F**) cells incubated with the Au and Au–Fe NPs for 72 h (*n* = 2). (**G**) IC50 values obtained from the viability curves.

The MTT assay, a standard methodology used in screening drugs and compounds, was the method of choice to assess the in vitro biocompatibility of the Au and Au–Fe NPs. From this test, the best candidates were chosen for further assessments. A relatively long incubation time of 72 h and an NP content up to 300 µg/mL were used as the appropriate conditions to stress the effect of cell exposure to the particles, because shorter times and lower concentrations may not be enough to show the effects of nanomaterials on cell health. Initially, normal (fibroblasts, BJ) and cancer (prostate carcinoma, PC3) cells were considered to be representative of healthy and tumor tissues, respectively. Fibroblasts are commonly used as a healthy cell line control, whereas prostate cancer cell lines such as PC3 are well-established and studied models, also for X-ray radiotherapy [26], and are desirable for considering the potential applications of the NPs as theragnostic agents. All of the Au and Au–Fe formulations were tested in these cell lines (Figure 2). The PEG-Au NPs had a high compatibility with the fibroblasts, and the PEG-Au–Fe n1 NPs reached a minimum viability of around 75% at 150 µg/mL, although they were less tolerated than the pure Au benchmark (Figure 2A). Interestingly, for concentrations below 50 µg/mL for the PEG-Au NPs, there was an increase in viability, which may indicate an increase in metabolic activity.

The biocompatibility changed dramatically when the PEG-Au and PEG-Au–Fe n1 NPs were embedded in PLGA, thus increasing their overall size (Figure 2A). It is interesting that the cytotoxicity is equivalent for the PLGA-PEG-Au and PLGA-PEG-Au–Fe NPs, suggesting that the polymeric coating is responsible for the main effect on the cell response. The other PEG-Au–Fe NPs, n2 to n5, were more toxic for the fibroblasts (Figure 2B), with viabilities below 50% at NP concentrations of 75 µg/mL. However, the TEOS-coated PEG-Au–Fe NPs exhibited the highest toxicity, with viabilities below 25% at NP concentrations of 75 µg/mL (Figure 2C).

In the PC3 cells, while the PEG-Au NPs confirmed their biocompatibility and the increase in cell metabolic activity below 50 µg/mL, the PEG-Au–Fe n1 NPs were remarkably toxic (Figure 2D), with viabilities below 40% already at NP concentrations of 75 µg/mL. After the PLGA coating, the toxicity of the PEG-Au NPs increased as observed in the fibroblasts, while the PEG-Au–Fe NPs maintained at the same high toxicity of the pristine nanoalloys. The n2 to n5 PEG-Au–Fe NPs (Figure 2E) and the a and b TEOS-PEG-Au–Fe NPs (Figure 2F) were again cytotoxic for the PC3 cells and more toxic than the n1 formulation, with the sole exception of the n5 sample. The toxicity of the Au–Fe formulations was generally lower for the fibroblasts.

To further compare the formulations, the IC50 was calculated as the concentration that results in a 50% loss in viability, shown in Figure 2G. The reliability of the IC50 value is higher when the viability curve ranges from 100% to 0%, because this allows the best fitting interval. However, this is not possible in the case of the NPs samples with limited cytotoxicity. Nonetheless, the obtained IC50 values confirm the specificity for tumor PC3 cells compared to fibroblasts of the PEG-Au–Fe n1 NPs and of the other Au–Fe NP samples with higher tolerability, while the Au formulations clearly have a higher IC50.

**Figure 3:**
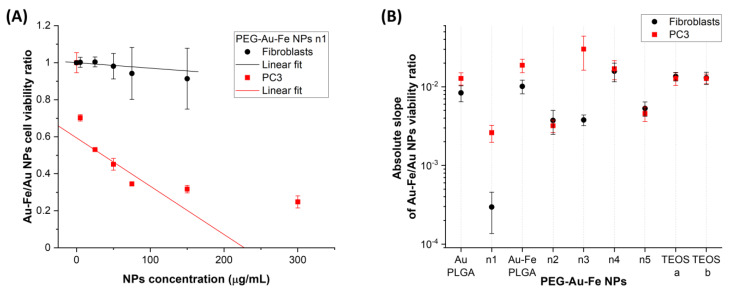
(**A**) Plot of the viability of fibroblasts or PC3 cells incubated with PEG-Au–Fe n1 NPs divide for that of the same cell types incubated with PEG-Au NPs. (**B**) Absolute slopes obtained from the linear fit of the relative cell viability between all samples and the benchmark PEG-Au NPs. A lower slope indicates similar viability to the biocompatible PEG-Au benchmark, thus higher biocompatibility.

To sum up the results in the two cell types and overcome the limitation of IC50 calculation, another empirical approach was adopted consisting of a comparison of the various NPs formulations to the PEG-Au biocompatible benchmark via the ratio of the respective cell viabilities, as shown in Figure 3A for the PEG-Au–Fe n1 NPs in the PC3 and fibroblast cells. Then, the absolute slopes of the linear interpolations were compared for the various formulations (Figure 3B), with larger values corresponding to a higher toxicity compared to the PEG-Au reference. The linear fit for this set of data was performed by fixing the intercept at 1, since this corresponds to the control conditions with no NPs, where the viability must be equal. Although this approach is useful only for a qualitative comparison, because the ratio of the dose–response curve is not necessarily linear, it allows for an immediate screening of the cell response to the various formulations. From Figure 3B, it is immediately appreciable that the PLGA coating increased the toxicity of the PEG-Au NPs in both cell lines, as it happened also for the PLGA-PEG-Au–Fe n1 NPs in the fibroblasts. It is also evident that the spread between the biocompatibility in the fibroblasts and the PC3 cells is the largest for the n1 and n3 PEG-Au–Fe NP samples; however, only the n1 maintains good biocompatibility in the non-cancerous cells. The other Au–Fe NPs have similar slopes in the normal and cancer cells, although with a generally higher toxicity in the latter as observed previously. Hence, the comparison of the slopes of the relative viability in fibroblasts and PC3 cells confirms that healthy cells are more resistant than the cancer ones when iron is introduced together with gold as nanoparticles.

**Figure 4:**
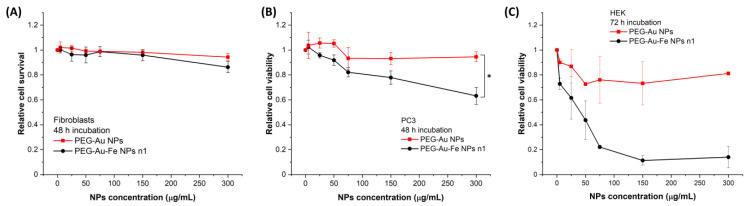
Cell viability results for fibroblasts (**A**) and PC3 (**B**) cells incubated for 48 h and HEK (**C**) cells incubated for 72 h with PEG-Au and PEG-Au–Fe n1 samples (*n* = 3). The symbol * represents a statistical difference (*p* < 0.05).

From this analysis, the PEG-Au–Fe n1 NPs emerged as the most promising formulation for further theragnostic applications. Thus, these NPs were tested also for a shorter incubation time of 48 h, and exhibited high compatibility with fibroblasts and viabilities close to 100%, even at the highest NP concentration (Figure 4A). In the PC3 cells, the viability of the PEG-Au NPs was again like that in the fibroblasts (Figure 4B), but the PEG-Au–Fe n1 NPs confirmed their greater toxicity in the tumor cells, with a viability close to 60% at a concentration of 300 µg/mL. The NPs were also tested for 72 h of incubation in human embryonic non-tumoral kidney (HEK) cells, which have a proliferation rate that is higher compared to fibroblasts and similar to PC3 cells. In the HEK cells, we observed a generally higher sensitivity to the presence of NPs, with high toxicity regarding the PEG-Au–Fe n1 NPs (Figure 4C) and a limited reduction in viability with the PEG-Au NPs at all concentrations tested up to 300 µg/mL.

**Figure 5:**
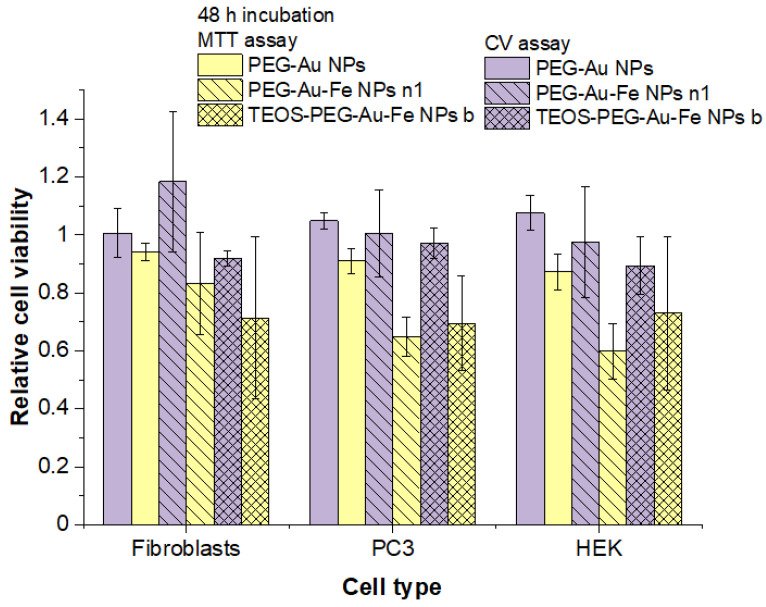
Comparative viability results from the MTT and CV assays for fibroblasts, PC3 and HEK cells incubated for 48 h with PEG-Au, PEG-Au–Fe n1 and TEOS-PEG-Au–Fe b samples (*n* = 3).

Since the MTT assay is an indirect test that measures metabolic activity, crystal violet (CV) staining and MTT assays were performed in parallel to verify if the results of the MTT tests were strictly due to a loss in viability. In fact, the CV binds to proteins and DNA and indirectly measures viability by relying on the detachment of dead cells from adherent cells in the culture plates [43]. For this analysis, the most biocompatible PEG-Au and PEG-Au–Fe n1 NPs were incubated at a concentration of 150 µg/mL; the most toxic formulation (TEOS-PEG-Au–Fe b) was also used, but at a lower concentration of 50 µg/mL, all for 48 h. Results are shown in Figure 5 in terms of means relative to the control with no NPs. Even though there is no significant difference within error among the MTT and CV results, we observed a systematic trend of higher values for the CV groups in contrast to those for the MTT for all NPs. This suggests that part of the toxicity seen in previous tests at 72 and 48 h is due to a reduction in metabolic viability instead of cell death only. This is in agreement with the absence of clear morphological differences after incubation (and washing of the wells) among the cells treated with the different NP samples. Even when the toxicity of the NPs was high, only a reduction in the number of cells was observed.

### 3.3. Origin of NPs Toxicity: ROS or Ferroptosis?

**Figure 6:**
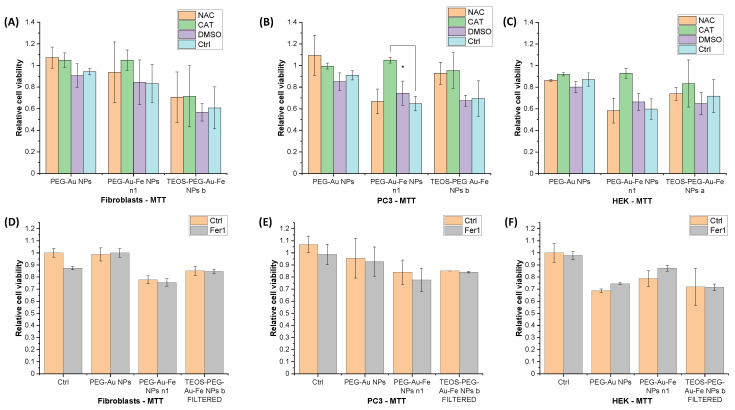
Effects of ROS scavengers NAC, CAT and DMSO ((**A**): fibroblasts, (**B**): PC3, (**C**): HEK cells) and ferroptosis inhibitor Fer1 ((**D**): fibroblasts, (**E**): PC3 cells, (**F**): HEK cells) on the viabilities of cells incubated for 48 h with PEG-Au, PEG-Au–Fe n1 and TEOS-PEG-Au–Fe b samples. The symbol * represents a statistical difference (*p* < 0.05). Note that the TEOS-PEG-Au–Fe b sample of the Fer1 test was filtered with a 200 nm filter to verify the effect of the removal of larger agglomerates on cell viability and ferroptosis.

To evaluate the role of ROS on the toxicity of the nanoparticles, the cells were incubated with different ROS scavengers prior to the addition of the NPs: catalase (CAT) (a scavenger of H_2_O_2_) [44,45,46], DMSO (a scavenger of OH) [47] and NAC (a broad ROS scavenger) [48]. The cell viability was analyzed after 48 h incubation with PEG-Au NPs and PEG-Au–Fe NPs n1 at 150 µg/mL and TEOS-PEG-Au–Fe b NPs at 50 µg/mL. The viabilities (Figure 6) were calculated relatively to the no-NPs control, with and without the scavengers. In fact, the viabilities of cells with NAC, CAT or DMSO and without NPs were in the 75–85% range of the cells alone. As shown in Figure 6A–C, for the PEG-Au NPs, which are the nontoxic benchmark, the presence of ROS scavengers is associated with a slight decrease in the viability of the PC3 cells and fibroblasts, without altering the results for the HEK cells. The addition of CAT resulted in higher viabilities for all cell types incubated with the PEG-Au–Fe n1 NPs. Noticeably, the difference among the control (no scavenger) and the other scavengers is larger in the PC3 and HEK cells, which were more affected by the Au–Fe NPs. Therefore, the toxicity of the PEG-Au–Fe NPs is due to the increase in hydrogen peroxide. The results for the TEOS-PEG-Au–Fe b NPs indicate that both NAC and CAT are associated with higher viabilities in all of the cell lines, although the difference is within error for the fibroblasts and HEK cells.

Since the Au–Fe NPs are known to undergo degradation over time in biological fluids, potentially providing free iron ions after the NPs are uptaken in cells, the occurrence of ferroptosis was investigated as another possible mechanism contributing to the toxicity of the nanoalloy samples. To this purpose, the widely used ferroptosis inhibitor ferrostatin-1 (Fer-1) was added to the wells, along with the nanoparticles, in a co-incubation of 48 h using a NP concentration of 50 µg/mL. In the results presented in Figure 6D–F, we observe that, even though the NPs toxicity was not high by itself, the addition of Fer-1 was not associated with any significant change in viability. The exclusion of ferroptosis as the main mechanism leading to NPs toxicity agrees with the nearly complete recovery of vitality observed in the cells treated with CAT.

## 4. Discussion

The in vitro biocompatibility of PEG-Au and PEG-Au–Fe NPs with various formulations was investigated, evidencing the different behavior of gold nanoalloys with iron compared to the pure gold benchmark. Importantly for directing the synthesis of the NPs, the embedding of NPs into PLGA or TEOS matrixes was associated with lower cell tolerability. This is associated with larger NPs agglomerates, which have a different interaction with the cell surface. Therefore, a further test was performed to verify the role of large agglomerates of NPs, by filtering the most toxic Au–Fe NP formulation (TEOS-PEG-Au–Fe b) with a 200 nm pore size filter before incubation with cells. The filtration is associated with a decrease in the absorbance in the near infrared range (Figure 1C) and to the recovery of NPs biocompatibility to the same level of the most tolerated formulation (PEG-Au–Fe n1, see Figure 6D). Overall, this test confirmed the importance of reducing the size of the NPs below the 200 nm threshold to obtain good in vitro tolerability.

To obtain further confirmation that the hydrodynamic size of the NPs is a relevant factor influencing their cytotoxicity, additional characterizations were performed on the two Au–Fe samples with low (PEG-Au–Fe n1) and high (TEOS-PEG-Au–Fe b) cytotoxicities, and comparing them to the PEG-Au NPs reference. The analysis showed no particular differences in the Au–Fe NPs’ structure (Figure S1) and coating (Figure S2), despite the presence of the silica layer in the TEOS-PEG-Au–Fe b samples. The z-potential of the Au–Fe samples was also similar (Figure S3A), whereas only the hydrodynamic size was remarkably different (Figure S3B). The hydrodynamic size of the PEG-Au NPs is comparable to that of the most biocompatible Au–Fe sample, as well as to that of the filtered TEOS-PEG-Au–Fe b sample (Figure S3B), which exhibited comparable toxicity to the PEG-Au–Fe n1 NPs, thus indicating that the silica coating is not responsible for a different cell tolerability.

Even in the absence of TEOS or PLGA, some PEG-Au–Fe NPs formulations displayed a very high toxicity in normal cells, while others were very well tolerated. The toxicities of the Au and Au–Fe formulations were compared through the slopes of the linear fitting of the dose–response curves, in order to highlight the differences due to the presence of iron in the gold nanostructures, as well as to stress the differences in toxicity in the PC3 cells versus the fibroblasts.

The cell viabilities for the less-toxic PEG-Au–Fe n1 NPs are equivalent to those previously reported for other iron alloys [49,50]. In the HeLa cells, toxicities below 20% and even at 200 µg/mL FePt NPs were measured, but for a short incubation time of 24 h, and similar results were observed with Fe–Ni nanosystems [49,50]. Viabilities above 80% for Au–Fe NPs tested in HeLa cells at concentrations close to 5 µM were measured for incubation times of 4 h [51]. The PEG-Au–Fe n1 NPs also displayed a remarkable difference in their cytotoxic potential when added to PC3 or fibroblast cells, indicating a potential selectivity to cancer cells that can be exploited for therapeutic purposes. Interestingly, when comparing the IC50 values for the PC3 and fibroblast cells (Figure 2G), the value for the PEG-Au–Fe n1 sample is sensibly higher in fibroblasts than in PC3, while the opposite is observed for the PEG-Au NPs, suggesting that the selectivity for the prostate cancer cells is proper for the Au–Fe nanoalloys.

It is important to note that the cell viability observed in our study was indirectly measured with the MTT assay, so it may be influenced by several factors involved in the cellular response to the NPs. Most importantly, marked changes in mitochondrial activity can result in significant changes in MTT values, although the number of viable cells remains constant [52]. For this reason, the comparative test using the CV assay was performed. The viability measured with the CV assay was generally higher, providing evidence that the toxicity caused by the nanoparticles is partially due a reduction in metabolic activity instead of immediate cell death. Iron-based material may release free iron, inducing iron dyshomeostasis, i.e., iron overload that leads to abundant ROS generation by Fenton reactions and, ultimately, catastrophic oxidative stress [53]. ROS may induce general damage to the cell or specifically trigger ferroptosis by lipid peroxidation, with consequent membrane impairment, lysosome dysfunction and mitochondrial damage [53].

To further investigate these mechanisms, ROS scavengers were used for their cytoprotective effect. It should be noted, however, that an increase in cell viability when the scavengers are present does not necessarily indicate a direct action on the ROS produced by the toxic agents. The antioxidant effect of scavengers is often due to fueling enzymatic cellular pathways [54,55,56].

Nevertheless, these experiments provide important information about the oxidative phenomena due to an increase in ROS production after incubation with the NPs. In the case of the Au–Fe NPs, we observed a protective effect only for CAT, which indicates a prominent role for hydrogen peroxide. The CAT enzyme uses iron as a cofactor in the reduction of H_2_O_2_ to water and molecular oxygen [57], so one possibility is that Au–Fe NPs facilitate the activity of CAT. The role of iron compounds in the activity of CAT is not yet well-identified in literature, but a study in a different biological model (Tubifex) showed that a mixture of Cd(II) and Fe(II) increases CAT activity in vivo after 48 h [58]. Excess dietary iron in rats, however, showed no increase in CAT activity [59]. Hence, additional investigations are required to understand the relationship between the CAT enzyme and Au–Fe alloys in vitro and in vivo. Concerning ferroptosis, no evidence of its occurrence was found in our experiment that added Fer-1, despite the Au–Fe NPs being biodegradable and releasing free iron ions over time.

On the other hand, we observed that the toxicity of Au–Fe NPs formulations was higher in PC3 cells compared to the fibroblasts, which can indicate a level of specificity that is desirable in treatment applications since it can achieve a curative response from the tumor while sparing the healthy tissues. Such selectivity can be linked to the high proliferation rate and metabolism typical of tumor cells, and this was confirmed in the fast-growing HEK cells, which were shown to be sensitive to Au–Fe NPs. Thus, the screening of Au–Fe NPs allowed for an understanding and selection of the most suitable candidates for a given application. Systemic administration of the NPs may exploit the enhanced permeability and retention effect, but a certain toxicity may arise in rapidly duplicating healthy cells, like blood and liver cells. This issue may be mitigated by the local delivery of NPs, which can be compatible and even more effective for testing the more toxic formulations. For instance, local delivery of nanoparticles is expected to allow the maximum uptake of the NPs in tumors, and can be performed via NP-loaded implants made from biodegradable polymers [60] or local injection [3,61,62,63].

## 5. Conclusions

In this study, we advanced the investigation of Au and Au–Fe alloy NPs in terms of their toxicities and mechanisms in vitro. We demonstrated that the PEG-Au and PEG-Au–Fe (formulation n1) NPs are well tolerated in fibroblasts. The cytotoxicity of the PEG-Au NPs was maintained also in PC3 and HEK cells, where the Au–Fe NPs were selectively more toxic. The agglomeration of Au and Au–Fe NPs by encapsulation in PLGA or embedding in TEOS were always associated with a decrease in the biocompatibility. The toxicity was reversible through the addition of the antioxidant catalase, and can be associated with the increased production of hydrogen peroxide due to the Fenton reaction in the presence of iron, which is released in solution due to the biodegradability of the Au–Fe nanoalloys. However, the toxicity was not directly related to ferroptosis. These results are crucial to further advance theragnostic applications of these nanosystems as contrast agents and radiosensitizers. Moreover, the investigation of the toxicity of NPs and their mechanisms are of general interest for the design of new nanomedicines based on Au and Fe.

## Data Availability

Data are available from the corresponding author upon reasonable request.

## Supplementary Materials

The following supporting information can be downloaded at: https://www.mdpi.com/article/10.3390/nano13233044/s1, Figure S1: XRD of NPs, Figure S2: FTIR of NPs, Figure S3: Z-potential and DLS of NPs.

## References

[1] Shi J., Kantoff P.W., Wooster R., Farokhzad O.C. (2017). Cancer Nanomedicine: Progress, Challenges and Opportunities. Nat. Rev. Cancer.

[2] Patra J.K., Das G., Fraceto L.F., Campos E.V.R., Rodriguez-Torres M.D.P., Acosta-Torres L.S., Diaz-Torres L.A., Grillo R., Swamy M.K., Sharma S. (2018). Nano Based Drug Delivery Systems: Recent Developments and Future Prospects. J. Nanobiotechnol..

[3] Yun W.S., Kim J., Lim D.K., Kim D.H., Jeon S.I., Kim K. (2023). Recent Studies and Progress in the Intratumoral Administration of Nano-Sized Drug Delivery Systems. Nanomaterials.

[4] Jiang Q., Yu L., Chen Y. (2023). Engineering Self-Assembled Nanomedicines Composed of Clinically Approved Medicines for Enhanced Tumor Nanotherapy. Nanomaterials.

[5] Kabashin A.V., Singh A., Swihart M.T., Zavestovskaya I.N., Prasad P.N. (2019). Laser-Processed Nanosilicon: A Multifunctional Nanomaterial for Energy and Healthcare. ACS Nano.

[6] Hamidu A., Pitt W.G., Husseini G.A. (2023). Recent Breakthroughs in Using Quantum Dots for Cancer Imaging and Drug Delivery Purposes. Nanomaterials.

[7] García A., Cámara J.A., Boullosa A.M., Gustà M.F., Mondragón L., Schwartz S., Casals E., Abasolo I., Bastús N.G., Puntes V. (2023). Nanoceria as Safe Contrast Agents for X-ray CT Imaging. Nanomaterials.

[8] Lopes T.S., Alves G.G., Pereira M.R., Granjeiro J.M., Leite P.E.C. (2019). Advances and Potential Application of Gold Nanoparticles in Nanomedicine. J. Cell. Biochem..

[9] Daniel M.C., Astruc D. (2004). Gold Nanoparticles: Assembly, Supramolecular Chemistry, Quantum-Size-Related Properties, and Applications toward Biology, Catalysis, and Nanotechnology. Chem. Rev..

[10] Hu Q., Fang Z., Ge J., Li H. (2022). Nanotechnology for Cardiovascular Diseases. Innovation.

[11] Sivasubramanian M., Chu C.H., Hsia Y., Chen N.T., Cai M.T., Tew L.S., Chuang Y.C., Chen C.T., Aydogan B., De Liao L. (2023). Illuminating and Radiosensitizing Tumors with 2DG-Bound Gold-Based Nanomedicine for Targeted CT Imaging and Therapy. Nanomaterials.

[12] Alhussan A., Jackson N., Calisin R., Morgan J., Beckham W., Chithrani D.B. (2023). Utilizing Gold Nanoparticles as Prospective Radiosensitizers in 3D Radioresistant Pancreatic Co-Culture Model. Int. J. Mol. Sci..

[13] Laprise-Pelletier M., Simão T., Fortin M.-A. (2018). Gold Nanoparticles in Radiotherapy and Recent Progress in Nanobrachytherapy. Adv. Healthc. Mater..

[14] Adam A., Mertz D. (2023). Iron Oxide@Mesoporous Silica Core-Shell Nanoparticles as Multimodal Platforms for Magnetic Resonance Imaging, Magnetic Hyperthermia, Near-Infrared Light Photothermia, and Drug Delivery. Nanomaterials.

[15] Arsalani S., Arsalani S., Isikawa M., Guidelli E.J., Mazon E.E., Ramos A.P., Bakuzis A., Pavan T.Z., Baffa O., Carneiro A.A.O. (2023). Hybrid Nanoparticles of Citrate-Coated Manganese Ferrite and Gold Nanorods in Magneto-Optical Imaging and Thermal Therapy. Nanomaterials.

[16] Farinha P., Coelho J.M.P., Reis C.P., Gaspar M.M. (2021). A Comprehensive Updated Review on Magnetic Nanoparticles in Diagnostics. Nanomaterials.

[17] Ezealigo B.N., Ezealigo U.S., Ighodalo K.I., Ezema F.I. (2022). Iron Oxide Nanoparticles: Current and Future Applications in Nanomedicine. Fundam. Ind. Appl. Magn. Nanoparticles.

[18] Wang Z., Qiao R., Tang N., Lu Z., Wang H., Zhang Z., Xue X., Huang Z., Zhang S., Zhang G. (2017). Active Targeting Theranostic Iron Oxide Nanoparticles for MRI and Magnetic Resonance-Guided Focused Ultrasound Ablation of Lung Cancer. Biomaterials.

[19] Torresan V., Forrer D., Guadagnini A., Badocco D., Pastore P., Casarin M., Selloni A., Coral D., Ceolin M., Fernández van Raap M.B. (2020). 4D Multimodal Nanomedicines Made of Nonequilibrium Au-Fe Alloy Nanoparticles. ACS Nano.

[20] Li B., Lane L.A. (2019). Probing the Biological Obstacles of Nanomedicine with Gold Nanoparticles. Wiley Interdiscip. Rev. Nanomed. Nanobiotechnol..

[21] Avramescu M.L., Chénier M., Beauchemin S., Rasmussen P. (2023). Dissolution Behaviour of Metal-Oxide Nanomaterials in Various Biological Media. Nanomaterials.

[22] Luo D., Wang X., Zeng S., Ramamurthy G., Burda C., Basilion J.P. (2019). Prostate-Specific Membrane Antigen Targeted Gold Nanoparticles for Prostate Cancer Radiotherapy: Does Size Matter for Targeted Particles?. Chem. Sci..

[23] Schuemann J., Bagley A.F., Berbeco R., Bromma K., Butterworth K.T., Byrne H.L., Chithrani B.D., Cho S.H., Cook J.R., Favaudon V. (2020). Roadmap for Metal Nanoparticles in Radiation Therapy: Current Status, Translational Challenges, and Future Directions. Phys. Med. Biol..

[24] Bravin C., Amendola V. (2020). Wide Range Detection of C-Reactive Protein with a Homogeneous Immunofluorimetric Assay Based on Cooperative Fluorescence Quenching Assisted by Gold Nanoparticles. Biosens. Bioelectron..

[25] Amendola V., Guadagnini A., Agnoli S., Badocco D., Pastore P., Fracasso G., Gerosa M., Vurro F., Busato A., Marzola P. (2021). Polymer-Coated Silver-Iron Nanoparticles as Efficient and Biodegradable MRI Contrast Agents. J. Colloid Interface Sci..

[26] Scaramuzza S., de Faria C.M.G., Coviello V., Forrer D., Artiglia L., Badocco D., Pastore P., Ghigna P., Postuma I., Cansolino L. (2023). A Laser Synthesis Route to Boron-Doped Gold Nanoparticles Designed for X-Ray Radiotherapy and Boron Neutron Capture Therapy Assisted by CT Imaging. Adv. Funct. Mater..

[27] Soares S., Sousa J., Pais A., Vitorino C. (2018). Nanomedicine: Principles, Properties, and Regulatory Issues. Front. Chem..

[28] Reda M., Bagley A.F., Zaidan H.Y., Yantasee W. (2020). Augmenting the Therapeutic Window of Radiotherapy: A Perspective on Molecularly Targeted Therapies and Nanomaterials. Radiother. Oncol..

[29] Laux P., Riebeling C., Booth A.M., Brain J.D., Brunner J., Cerrillo C., Creutzenberg O., Estrela-Lopis I., Gebel T., Johanson G. (2017). Biokinetics of Nanomaterials: The Role of Biopersistence. NanoImpact.

[30] Sharma S., Parmar A., Kori S., Sandhir R. (2016). PLGA-Based Nanoparticles: A New Paradigm in Biomedical Applications. TrAC Trends Anal. Chem..

[31] Amendola V., Scaramuzza S., Litti L., Meneghetti M., Zuccolotto G., Rosato A., Nicolato E., Marzola P., Fracasso G., Anselmi C. (2014). Magneto-Plasmonic Au-Fe Alloy Nanoparticles Designed for Multimodal SERS-MRI-CT Imaging. Small.

[32] AAT Bioquest Inc. Quest Graph^TM^ IC50 Calculator. https://www.aatbio.com/tools/ic50-calculator.

[33] Popov A.A., Swiatkowska-Warkocka Z., Marszalek M., Tselikov G., Zelepukin I.V., Al-Kattan A., Deyev S.M., Klimentov S.M., Itina T.E., Kabashin A.V. (2022). Laser-Ablative Synthesis of Ultrapure Magneto-Plasmonic Core-Satellite Nanocomposites for Biomedical Applications. Nanomaterials.

[34] Torresan V., Guadagnini A., Badocco D., Pastore P., Muñoz Medina G.A., Fernàndez van Raap M.B., Postuma I., Bortolussi S., Bekić M., Čolić M. (2020). Biocompatible Iron–Boron Nanoparticles Designed for Neutron Capture Therapy Guided by Magnetic Resonance Imaging. Adv. Healthc. Mater..

[35] Gurbatov S., Puzikov V., Modin E., Shevlyagin A., Gerasimenko A., Mitsai E., Kulinich S.A., Kuchmizhak A. (2022). Ag-Decorated Si Microspheres Produced by Laser Ablation in Liquid: All-in-One Temperature-Feedback SERS-Based Platform for Nanosensing. Materials.

[36] Fromme T., Tintrop L.K., Reichenberger S., Schmidt T.C., Barcikowski S. (2023). Impact of Chemical and Physical Properties of Organic Solvents on the Gas and Hydrogen Formation during Laser Synthesis of Gold Nanoparticles. ChemPhysChem.

[37] Hesabizadeh T., Sung K., Park M., Foley S., Paredes A., Blissett S., Guisbiers G. (2023). Synthesis of Antibacterial Copper Oxide Nanoparticles by Pulsed Laser Ablation in Liquids: Potential Application against Foodborne Pathogens. Nanomaterials.

[38] Crivellaro S., Guadagnini A., Arboleda D.M.D.M., Schinca D., Amendola V. (2019). A System for the Synthesis of Nanoparticles by Laser Ablation in Liquid That Is Remotely Controlled with PC or Smartphone. Rev. Sci. Instrum..

[39] Khairani I.Y., Mínguez-Vega G., Doñate-Buendía C., Gökce B. (2023). Green Nanoparticle Synthesis at Scale: A Perspective on Overcoming the Limits of Pulsed Laser Ablation in Liquids for High-Throughput Production. Phys. Chem. Chem. Phys..

[40] Kralj S., Makovec D., Čampelj S., Drofenik M. (2010). Producing Ultra-Thin Silica Coatings on Iron-Oxide Nanoparticles to Improve Their Surface Reactivity. J. Magn. Magn. Mater..

[41] Čampelj S., Makovec D., Drofenik M. (2009). Functionalization of Magnetic Nanoparticles with 3-Aminopropyl Silane. J. Magn. Magn. Mater..

[42] Ali Z., Andreassen J.-P., Bandyopadhyay S. (2023). Fine-Tuning of Particle Size and Morphology of Silica Coated Iron Oxide Nanoparticles. Ind. Eng. Chem. Res..

[43] Feoktistova M., Geserick P., Leverkus M. (2016). Crystal Violet Assay for Determining Viability of Cultured Cells. Cold Spring Harb. Protoc..

[44] Huang G., Chen H., Dong Y., Luo X., Yu H., Moore Z., Bey E.A., Boothman D.A., Gao J. (2013). Superparamagnetic Iron Oxide Nanoparticles: Amplifying ROS Stress to Improve Anticancer Drug Efficacy. Theranostics.

[45] Wang Y., Qi H., Liu Y., Duan C., Liu X., Xia T., Chen D., Piao H.L., Liu H.X. (2021). The Double-Edged Roles of ROS in Cancer Prevention and Therapy. Theranostics.

[46] Zhang H., Yin M., Huang L., Wang J., Gong L., Liu J., Sun B. (2017). Evaluation of the Cellular and Animal Models for the Study of Antioxidant Activity: A Review. J. Food Sci..

[47] Simunkova M., Barbierikova Z., Jomova K., Hudecova L., Lauro P., Alwasel S.H., Alhazza I., Rhodes C.J., Valko M. (2021). Antioxidant vs. Prooxidant Properties of the Flavonoid, Kaempferol, in the Presence of Cu(II) Ions: A ROS-Scavenging Activity, Fenton Reaction and DNA Damage Study. Int. J. Mol. Sci..

[48] Wang L., Wang Z., Li X., Zhang Y., Yin M., Li J., Song H., Shi J., Ling D., Wang L. (2018). Deciphering Active Biocompatibility of Iron Oxide Nanoparticles from Their Intrinsic Antagonism. Nano Res..

[49] Chou S.W., Liu C.L., Liu T.M., Shen Y.F., Kuo L.C., Wu C.H., Hsieh T.Y., Wu P.C., Tsai M.R., Yang C.C. (2016). Infrared-Active Quadruple Contrast FePt Nanoparticles for Multiple Scale Molecular Imaging. Biomaterials.

[50] Yang H., Li X., Zhou H., Zhuang Y., Hu H., Wu H., Yang S. (2011). Monodisperse Water-Soluble Fe–Ni Nanoparticles for Magnetic Resonance Imaging. J. Alloys Compd..

[51] Sousa F., Sanavio B., Saccani A., Tang Y., Zucca I., Carney T.M., Mastropietro A., Jacob Silva P.H., Carney R.P., Schenk K. (2017). Superparamagnetic Nanoparticles as High Efficiency Magnetic Resonance Imaging T2 Contrast Agent. Bioconjugate Chem..

[52] Vega-Avila E., Pugsley M.K. (2011). An Overview of Colorimetric Assay Methods Used to Assess Survival or Proliferation of Mammalian Cells. Proc. West. Pharmacol. Soc..

[53] Zheng H., Jiang J., Xu S., Liu W., Xie Q., Cai X., Zhang J., Liu S., Li R. (2021). Nanoparticle-Induced Ferroptosis: Detection Methods, Mechanisms and Applications. Nanoscale.

[54] Kalyanaraman B. (2022). NAC, NAC, Knockin’ on Heaven’s Door: Interpreting the Mechanism of Action of N-Acetylcysteine in Tumor and Immune Cells. Redox Biol..

[55] Pedre B., Barayeu U., Ezeriņa D., Dick T.P. (2021). The Mechanism of Action of N-Acetylcysteine (NAC): The Emerging Role of H2S and Sulfane Sulfur Species. Pharmacol. Ther..

[56] Mlejnek P. (2022). Direct Interaction between N-Acetylcysteine and Cytotoxic Electrophile—An Overlooked In Vitro Mechanism of Protection. Antioxidants.

[57] Ighodaro O.M., Akinloye O.A. (2018). First Line Defence Antioxidants-Superoxide Dismutase (SOD), Catalase (CAT) and Glutathione Peroxidase (GPX): Their Fundamental Role in the Entire Antioxidant Defence Grid. Alex. J. Med..

[58] Chen T., Furst A., Chien P.K. (1994). The Effects of Cadmium and Iron on Catalase Activities in Tubifex. Int. J. Toxicol..

[59] Lee Y.H., Layman D.K., Bell R.R., Norton H.W. (1981). Response of Glutathione Peroxidase and Catalase to Excess Dietary Iron in Rats. J. Nutr..

[60] Boateng F., Ngwa W. (2019). Delivery of Nanoparticle-Based Radiosensitizers for Radiotherapy Applications. Int. J. Mol. Sci..

[61] Hu J., Dong Y., Ding L., Dong Y., Wu Z., Wang W., Shen M., Duan Y. (2019). Local Delivery of Arsenic Trioxide Nanoparticles for Hepatocellular Carcinoma Treatment. Signal Transduct. Target. Ther..

[62] Caddy G., Stebbing J., Wakefield G., Xu X.Y. (2022). Modelling of Nanoparticle Distribution in a Spherical Tumour during and Following Local Injection. Pharmaceutics.

[63] Chao Y., Chen G., Liang C., Xu J., Dong Z., Han X., Wang C., Liu Z. (2019). Iron Nanoparticles for Low-Power Local Magnetic Hyperthermia in Combination with Immune Checkpoint Blockade for Systemic Antitumor Therapy. Nano Lett..

