# Supplementary material for: Cytotoxicity of PEG-Coated Gold and Gold–Iron Alloy Nanoparticles: ROS or Ferroptosis?
Source: Nanomaterials (Basel). 2023 Nov 29;13(23):3044. doi: 10.3390/nano13233044 (PMC10708329; doi:10.3390/nano13233044)
Supplement: Supplementary file 1 [file nanomaterials-13-03044-s001.zip › nanomaterials-2700968-supplementary.pdf]

Supporting Information

Cytotoxicity of PEG-Coated Gold and Gold-Iron Alloy Nanoparticles: ROS or Ferroptosis?

Clara M.G. de Faria,[1] Michael Bissoli,[1] Riccardo Vago,[2] Antonello E. Spinelli,[3] Vincenzo Amendola[1]*

[1] Department of Chemical Sciences, University of Padova, Via Marzolo 1, Padova I-35131, Italy, claramaria.goncalvesdefaria@unipd.it; michael.bissoli@unipd.it
[2] Division of Experimental Oncology, Urological Research Institute, IRCCS San Raffaele Scientific Institute, Milan, Italy; vago.riccardo@hsr.it
[3] Experimental Imaging Center, IRCCS San Raffaele Scientific Institute, Milan, Italy, spinelli.antonello@hsr.it
* Correspondence: vincenzo.amendola@unipd.it

Index:

1. **XRD of PEG-Au, PEG-Au-Fe n1 and TEOS-PEG-Au-Fe b NPs**
2. **FTIR of PEG-Au, PEG-Au-Fe n1 and TEOS-PEG-Au-Fe b NPs**
3. **Z-potential and DLS of PEG-Au, PEG-Au-Fe n1 and TEOS-PEG-Au-Fe b NPs**

1. XRD of PEG-Au, PEG-Au-Fe n1 and TEOS-PEG-Au-Fe b NPs

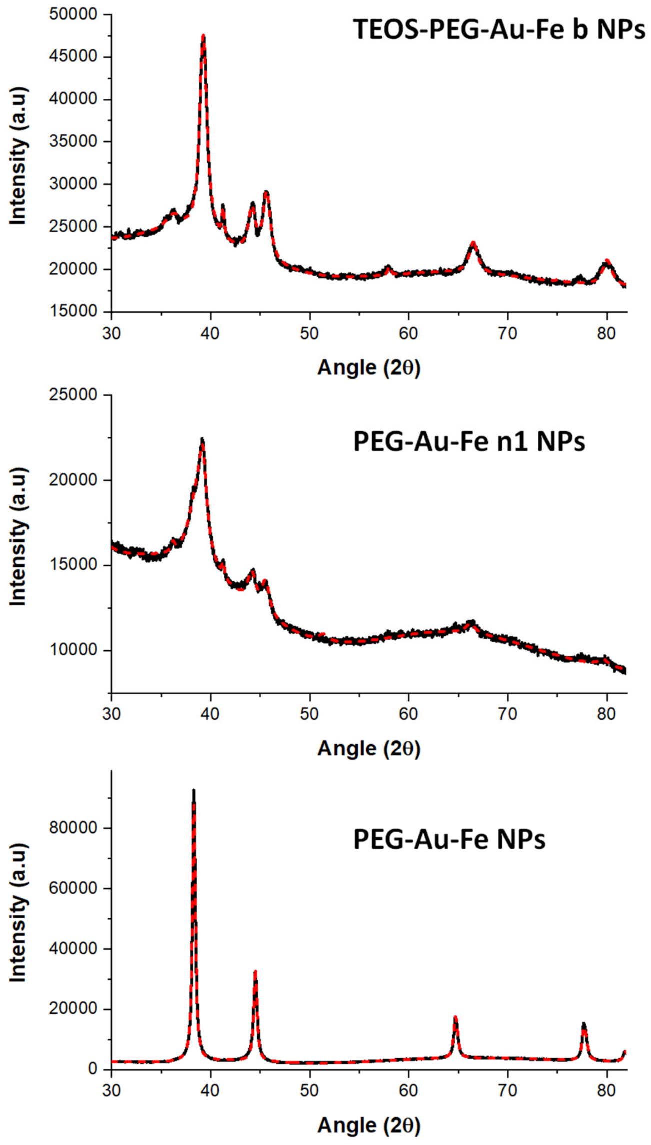

Figure S1. XRD pattern (continuous lines) and Rietveld refinement (dashed lines) of the PEG-Au, PEG-Au-Fe n1 and TEOS-PEG-Au-Fe b NPs samples. The Au NPs have the typical face centered cubic (FCC) lattice with a cell parameter in agreement with that of bulk gold (4.0775 ± 0.0001 Å). The Au-Fe NPs have the well-known disordered structure (large peaks) due to the metastability of the alloy, and two FCC components can be identified, one ascribable to an Au-rich alloy (cell parameter of 4.0011 ± 0.0022 Å for PEG-Au-Fe n1 and 3.9783 ± 0.0006 Å for TEOS-PEG-Au-Fe b) and another to iron-rich alloy (cell parameter 3.5703 ± 0.0020 Å for PEG-Au-Fe n1 and 3.5558 ± 0.0007 Å for TEOS-PEG-Au-Fe b). Therefore, the same phases are observed in the PEG-Au-Fe n1 and TEOS-PEG-Au-Fe b NPs samples.

2. FTIR of PEG-Au, PEG-Au-Fe n1 and TEOS-PEG-Au-Fe b NPs

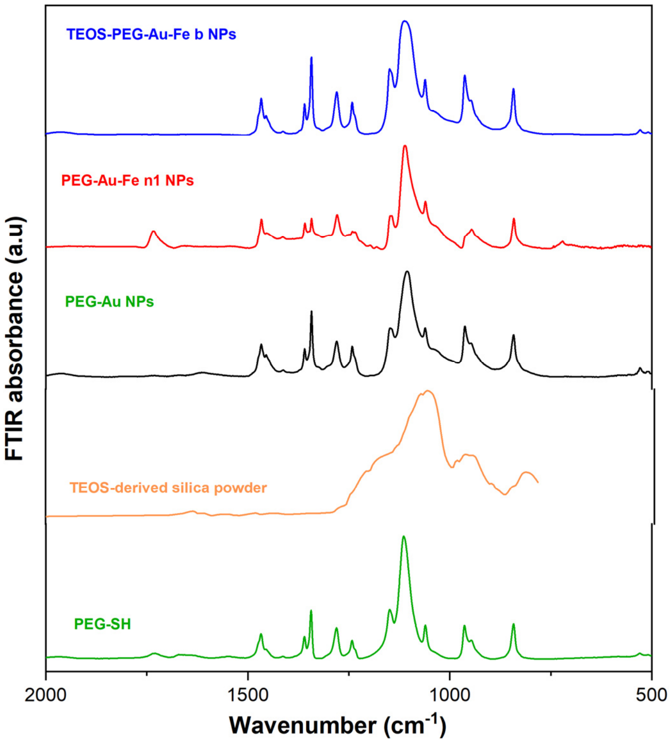

Figure S2. FTIR spectra of PEG-Au, PEG-Au-Fe n1 and TEOS-PEG-Au-Fe b NPs samples. In all cases, the peaks characteristic of PEG are well evident, indicating a comparable polymeric coating. The peaks of the TEOS-derived silica shell are difficult to identify below the intense PEG signals in the TEOS-PEG-Au-Fe b NPs sample.

3. Z-potential and DLS of PEG-Au, PEG-Au-Fe n1 and TEOS-PEG-Au-Fe b NPs

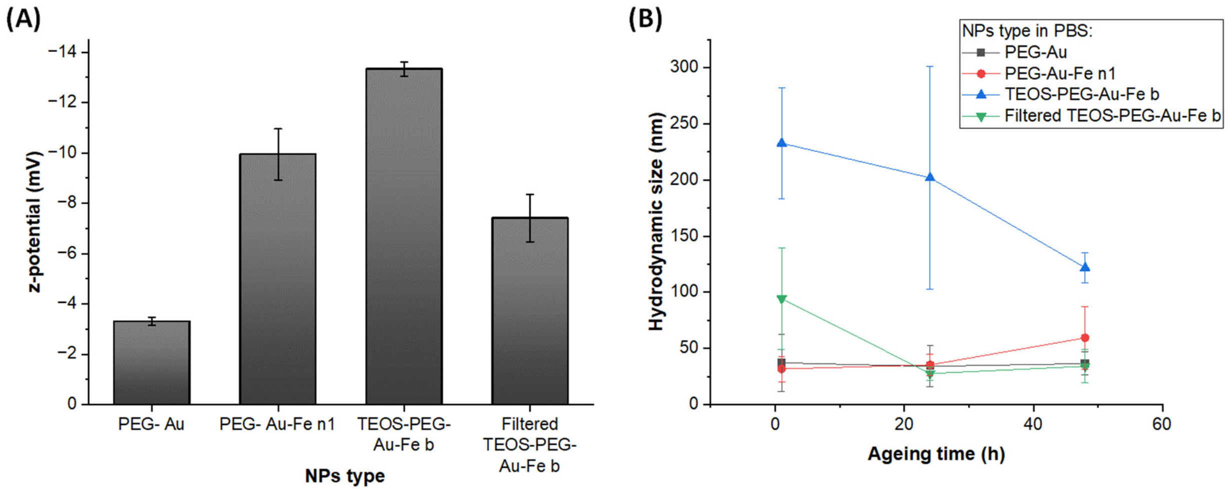

Figure S3. Z-potential (A) and DLS measured hydrodynamic size (B) of PEG-Au, PEG-Au-Fe n1 and TEOS-PEG-Au-Fe b NPs in phosphate buffered saline solution after ageing for 1, 24 and 48 h at 37 °C. The results for the TEOS-PEG-Au-Fe b NPs after filtration with a with a 200 nm pore size filter are also shown, indicating the size reduction to values similar to PEG-Au and PEG-Au-Fe n1 NPs. This is accompanied by a reduced cytotoxicity to values equivalent to the PEG-Au-Fe n1 NPs. Note the relatively low z-potential, suggesting the prevalence of a steric repulsion mechanism for the stability of the NPs.
